# Effect of Follicular Unit Extraction on the Donor Area

**Published:** 2018-05

**Authors:** Muhamamd Humayun Mohmand, Muhammad Ahmad

**Affiliations:** Plastic and Hair Restorative Surgery Department, Hair Transplant Institute, Islamabad, Pakistan

**Keywords:** Follicular unit extraction, Hair restoration, Donor

## Abstract

**BACKGROUND:**

Hair restoration surgery is one of the most commonly performed cosmetic surgery procedure in men. The main aim of the study was to know the effect of follicular unit extraction (FUE) follicular unit extraction (FUE) on donor area in terms of hair mass/density.

**METHODS:**

Ten male patients undergoing hair restoration by FUE were included. In each patient, ten boxes of 1 cm^2^ each were marked. The first box was marked in the midline and the 2^nd^ and 3^rd^ boxes were marked about 3 cm from the midline. Another two boxes, each of 1 cm^2^ were also marked at the distance of 3 cm. Two boxes of 1 cm^2^ were marked on either side. The extraction was performed using 0.9 mm punch. The number of extracted hair were counted.

**RESULTS:**

The mean age of the patients was 31.7 years. The average hair count in the donor area was 154.76 hair per cm^2^. The extracted hair count was 54.85 hair per cm^2^ which was about 35.44% of the total donor density (range: 28.9-42.8%). The graft to hair ratio in the extracted follicular units was 1:2.3 (range: 1:1.65-1:2.75).

**CONCLUSION:**

As the donor density varies, the FUE should be performed with caution.

## INTRODUCTION

Hair restoration surgery is one of the most commonly performed cosmetic surgery procedure in men. More than sixty percent of the male population experiences some degree of hair loss at some stage of their lives.^1^ There are mainly two types of surgical procedures performed namely the strip surgery and follicular unit extraction (FUE). The strip surgery, also known as follicular unit transplantation (FUT) or follicular unit strip surgery (FUSS). The second kind of surgery, FUE, involves the harvesting of follicular units from the donor area with the help of manual machines/instruments, motorized machines or robots. Over the last decade, FUE has gained more popularity due to certain commercial, technical and other factors.^1^

No study to date is available which could prove the superiority of one technique over the other. Both techniques are technically demanding, require thorough knowledge of the hair restoration process and the surgical expertise to carry out the procedure. Each have certain complications. The FUE technique is more popularized due to the fact of lack of linear scar but the total surface area of the scars is many times larger than the surface area of the scar of a strip surgery. Similarly, FUE technique allows harvesting of the follicular units from a larger area which is not possible with the strip surgery.^2^ The pros and cons of each procedure depend on the patient and surgical expertise of the surgeon.

As the hair loss is not static, the final phenotype is determined by the genes. The male hormones and the therapies used to alter the hair loss process.^3^ The density of the given area commonly keeps on decreasing with the advancing age. There is no study mentioned in the literature which determines the effect of FUE on the donor area. The main aim of the study was to know the effect of FUE on donor area in terms of hair mass/density. 

## MATERIALS AND METHODS

**Fig. 1:**
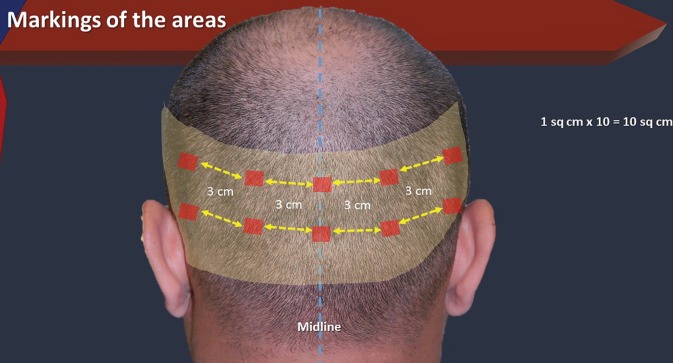
: Scheme of the study

The study was undertaken in a private hair restoration clinic. Ten male patients undergoing hair restoration by FUE were included in the study. Patients having a previous strip or FUE surgery were excluded. Detailed history and informed consent was obtained from all the patients. The preoperative photographs were taken. Donor area was marked within the safe limits. In each patient, ten boxes of 1 cm^2^ each were marked. The first box (A) was marked in the midline at the level of external occipital protuberance (Figure 1). 

The 2^nd^ and 3^rd^ boxes (B and C) were marked about 3 cm from the midline. Another two boxes (D and E), each of 1 cm^2^ were also marked at the distance of 3 cm from the outer border of the box B and C. About 3cm below the box A, another box of 1 cm^2^ was marked (F). Two boxes of 1cm^2^ each were marked on either side (G and I on one side and H and J on the other side) of the box F as described earlier with an intervening distance of 3 cm. All of these boxes were photographed using a macro lens. The hairs were counted manually in each of these boxes. The extraction was performed using 0.9 mm punch. The number of extracted hair were counted for each box in each patient. The boxes were photographed postoperatively. 

## RESULTS

**Fig. 2:**
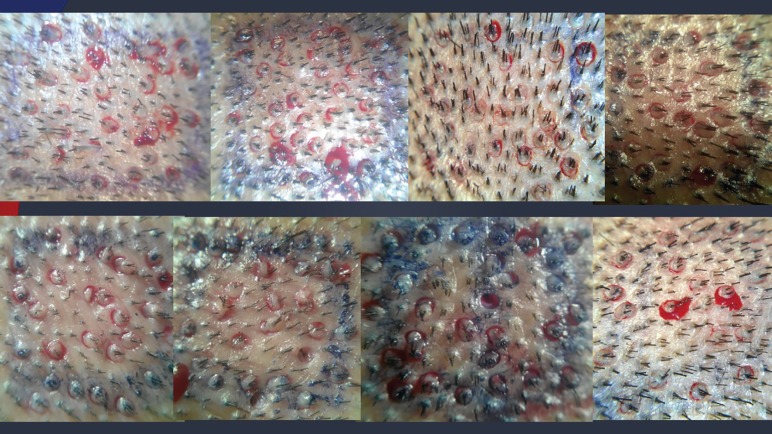
Extent of FUE

Ten patients were enrolled in the study. In each patient, the total area of 10 cm^2^ was included (total area of 100 cm^2^ in ten patients). The mean age of the patients was 31.7 years. The average hair count in the donor area was 154.76 hair per cm^2^ (Table 1). The 0.9 mm punch was used for extraction. The extracted hair count was 54.85 hair per cm^2^ which was about 35.44% of the total donor density (range: 28.9-42.8%). The graft to hair ratio in the extracted follicular units was 1:2.3 (range: 1:1.65-1:2.75) (Figure 2).

**Table 1:** Patients’ data

**No**	**Total hair count**	**Extracted hair count**	***%***	**Hair ratio Total: Extracted**	**Graft: Hair ratio**
1	1486	584	39.1	2.54:1	1:2.42
2	1563	651	41.7	2.4:1	1:2.37
3	1153	340	29.5	3.39:1	1:2.16
4	1782	762	42.8	2.34:1	1:2.08
5	1584	457	28.9	3.47:1	1:1.65
6	1793	671	37.4	2.67:1	1:2.56
7	1882	715	40.0	2.63:1	1:2.75
8	1536	515	33.5	2.98:1	1:2.15
9	1216	311	25.6	3.91:1	1:2.08
10	1481	479	32.3	3.10:1	1:2.30
Total	15476	5485	35.4	2.82:1	1:2.26

## DISCUSSION

Removing hair from the donor involves the expertise on the part of the surgeon. Over the last decade, there has been increased trend in FUE procedure by the physicians. Those patients who want to have short hair or who want to avoid linear scar, now can undergo hair restoration procedure. The donor area contains hair clustered into ‘follicular units’. The defining term for donor area remains the ‘hair density’. The donor density varies according to the age and race. For example, hair density in Caucasians is different from Asian or Africa.^4^^-^^6^

Both FUE and FUT reduce the donor area but the way of execution is different. FUT decreases the height of donor area, a part of it gets re-stretched by ‘stretch-back’ phenomenon.^7^ Whereas FUE reduces the overall density by ‘cherry-picking’. FUE is not a straight forward or simple procedure. It has a steep learning curve, need a physical stamina, patience, excellent hand-eyes coordination etc. It also requires diagnostic skill, aesthetic insight and sensitivity and expertize to deal with rare unexpected complications. Various side effects of FUE have been mentioned in the literature and this list is increasing every year due to larger number of procedures performed by novice surgeons. These side-effects include visible scarring, skin necrosis, cyst formation, neuralgia, hiccups etc.^8^^-^^10^

Although various surgeons’ experiences have been presented in the literature and conference with excellent results but no study is yet there to show the long term results after FUE procedure. The most important point in FUE surgery is the number of hair which is removed from the donor area. The transection rate plays an important role in the outcome of a hair restoration surgery by FUE.^11^ There are multiple factors which predict the success of the hair restoration surgery. The patient’s age, type of baldness, family history, environmental factors all play a vital role. As the hair loss is an ongoing process which never halts at any age, decrease in density in the donor area is also seen.^12^^,^^13^

In the study by Bernstein, the average graft to hair ratio was 1:2.72.^14^ All the studies done on FUE describe the removal/extraction of grafts or number of hair. None of these describes the effect on donor hair. Harvesting more than 3000 grafts in no doubt a great effort on the part of the surgeon but the question of hair density left behind and transection remains there. In a study by Avram using robotic FUE, the overall transection rate was 6.6 with a wide range from 0.4% to 32.1%.^15^ The more the number of grafts harvested, the more is the transection rate. On the other hand, a transection rate of 1.59% is mentioned in the study by Pathomvanich et al.^16^ A few techniques, e.g., use of Haber’s dissector and scissors tissue dissection, may have even lower transection rates in FUT surgery.^16^^,^^17^

Most of the published data on FUE indicate the inclusion of small areas in the studies. The authors are of the views that there are certain key limitation points which are not included. One of the most important point omitted is the ‘fatigue’ factor while harvesting larger number of FUs (normally more than 2500). Another equally important factor is the angle of hair exit. The angles become more acute on sides and below superior nuchal line which results in higher rate of transection. Similarly, hair angles also vary in different races, e.g., the chances of transection are higher in African than Caucasians. The authors also assume the transection rate mentioned in the many studies may not depict the real picture due to these important factors. The hidden transection rate is also another factor.^11^

Harvesting more grafts means removal of FUs beyond the limits of ‘safe donor area’ which can be a potentially ‘danger zone’ or strictly an ‘unsafe zone’ to harvest. This unsafe/danger zone may be affected as a result of ongoing aging hairless and male pattern baldness which would result in slightly-scars of FUE if f it is done in a young patient. Most of the studies indicate that patients undergoing FUE are less than 35 years. Another important point is the definition of ‘safe donor area’ which should be revised. The authors are conducting a study to review the ‘donor area’ in the male population above the age of 50 years. Thus the key point in FUE remain the donor area (safe zone or danger zone or unsafe zone) and the number of extracted hair. In younger patients with advanced baldness (type V or more), the FUs are harvested from the non-safe areas by FUE.^18^

Many surgeons suggest that about 50% of the donor density can be harvested.^19^ The removal of 50% donor density means the FUE procedure has resulted near the level of ‘watershed line’ for ‘visibly detecting the hair loss’. A 2^nd^ session of FUE procedure of even 20% will reduce the overall donor density to about 70%, which will be obvious to the naked eye. The ongoing aging hair loss will also be added and would cause gross baldness and the visibility of the scarring of FUE. Therefore, the number of grafts removed in FUE is not important, rather the number of hair removed are of the utmost importance. The patients having 20–30% of the actual density in the donor area would look odd as the donor density will be low showing the scalp. In the current study, about 35% of the donor hair were extracted. In a study by Beehner conducted to compare the results of FUE and FUT, the survival rate of FUs by FUT was 86% as compared to 61.4% by FUE after 14 months postoperatively.^20^

Many factors play a role in the ultimate survival including, storage solution and its temperature, out of body time, desiccation/dehydration etc. The boom of FUE is resulting mainly because of the advertisement done by the FUE machines marketing companies and mainly doctors trying not to learn the surgical techniques. As described by Rassman, a new breed of hair restorative surgeons is coming in the market who are trained only for FUE and probably have no knowledge about the ‘strip harvest surgery’ and cannot offer the treatment to a patient if needed.^21^ The ethical issue may arise if they would not refer to a colleague who can perform both FUT and FUE. Moreover, the surgeons may be replaced eventually, after sometime, by the technicians and robots etc. as it is more cost-effective. In order to do hair restoration surgery for advanced baldness, the maximization of grafts harvesting should be done. This procedure is advertised as a ‘profitable turnkey’ model for new revenue streams to the physician’s practice. The extra role of ‘technicians’ or ‘non-physicians’ in various steps of hair restoration seems improper and illegal.^21^

The so called ‘Turkey-phenomenon’ where one physician, not necessarily the surgeon, supervises the various simultaneous surgeries done by the technicians. In an uncontrolled FUE environment, the danger of donor depletion also remains there. Criticism on FUE sometimes is taken as if the critique is against FUE whereas FUE is actually a great addition in the armamentarium of a hair restorative surgeon. The only point is its judicious use in the patients. A state of the art about two decades age, is not acceptable now. The decision should be tailor-made. 

From the study, there are a few recommendations for FUE proposed by the authors. (i) FUE should be limited to less than 35% of total hair density in 1^st^ session and not more than 10-20% in 2^nd^ session; (ii) FUE sessions should be combined with FUT to maximize the number of grafts/hair in a given session; (iii) FUE should be done carefully during the learning curve and initially should be done under supervision; (iv) When the final procedure is done, the scar of the strip surgery should be repaired by FUE from scalp or body or by micropigmentation; and (v) FUE should not be perfumed in very young patients and if needed, only grafts from the safe zone should be harvested. Family history should be kept in mind. Therefore, we can conclude that as the donor density varies, the FUE should be performed with caution.

## CONFLICT OF INTEREST

The authors declare no conflict of interest.
